# A Case Report of Acute Respiratory Distress Syndrome From Cannabis and Amphetamine Use

**DOI:** 10.7759/cureus.50003

**Published:** 2023-12-05

**Authors:** Mohamed S Shehataa, Ahmed H Abdelfattah, Ahmed N Selim

**Affiliations:** 1 Critical Care Medicine, Cairo University, Cairo, EGY; 2 Critical Care Medicine, Ibn Sina National College for Medical Studies, Jeddah, SAU; 3 Internal Medicine, University of Kentucky College of Medicine, Lexington, USA; 4 Critical Care Medicine, Beni Suef University Hospital, Beni Suef, EGY

**Keywords:** acute respiratory distress syndrome (ards), drug-induced lung injury, amphetamine abuse, cannabis use, illicit drug

## Abstract

Illicit drug usage (IDU) is a big challenge in clinical practice, with increasing incidence in the last decades. Daily, clinicians encounter a wide variety of complications related to IDU. Common infections related to illicit drugs are infective endocarditis, abscesses, osteomyelitis, pneumonia, HIV, hepatitis C, and B. Other rare complications could happen like leukoencephalopathy, IDU-related lung injury, and acute respiratory distress syndrome (ARDS) which is a severe and potentially life-threatening condition characterized by the sudden onset of respiratory failure, often necessitating mechanical ventilation. While the most common etiologies of ARDS are related to infections and sepsis, there is emerging evidence that substance abuse can also be associated with the development of ARDS with unclear mechanisms. IDU-related lung injury is a rare entity with few cases reported in the literature. Its management usually involves supportive care, including mechanical ventilation, oxygen therapy, and close monitoring of fluid balance. We present a case of a 25-year-old male presented with ARDS and multiorgan failure related to methamphetamine and cannabis abuse.

## Introduction

Illicit drug usage (IDU) is a global health issue that is becoming increasingly prevalent. IDU-related lung injuries could present in different ways like uncommonly acute respiratory distress syndrome (ARDS) which is a life-threatening condition characterized by a damaged epithelial-endothelial barrier, leading to leakage of blood cells and triggering accumulation of inflammatory mediators into the lung interstitium and alveoli, which leads to a significant hypoxia [1]. Causes of ARDS include infections, trauma, pancreatitis, and inhalation of toxic substances [2]. Despite being rare, inhaled illicit drugs have been reported to cause ARDS in some patients with unknown mechanisms to date, often with a high mortality rate and unclear pathogenesis [3]. IDU has a variety of pulmonary effects, including short-term, potentially fatal illnesses as well as long-term, functional respiratory implications. Enhanced comprehension of their range and the associated mechanisms could contribute to better patient care [3].

## Case presentation

**Figure 1:**
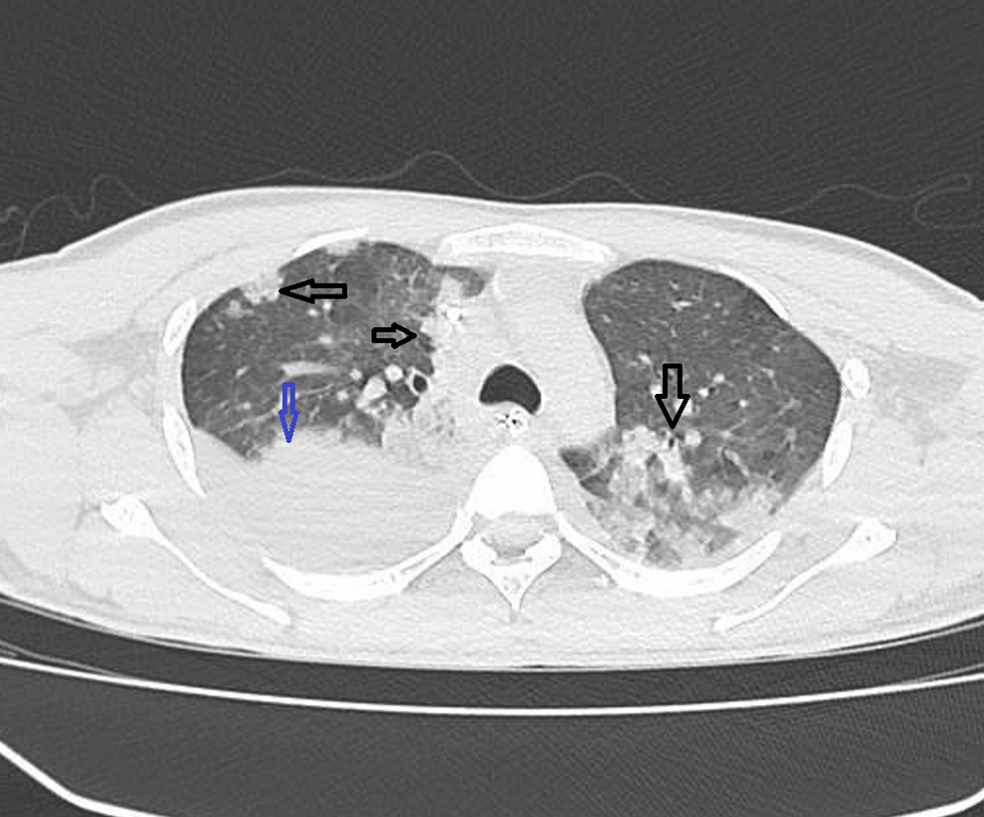
CT chest showing bilateral pulmonary infiltrates and right-sided effusion. The blue arrow shows right-side effusion and the black arrows show bilateral infiltrates.

We present a case of a 25-year-old male who presented to the emergency room (ER) with sudden onset of severe shortness of air, dyspnea, respiratory distress, and cyanosis for a few hours. There was no past medical history of chronic cardiac or pulmonary diseases. His medical history was significant only for IDU, which was confirmed by the family. The patient smoked methamphetamine and marijuana two days before the presentation. Physical examination revealed unstable vital signs with tachycardia of 140 beats per minute, tachypnea of 28 breaths per minute, oxygen saturation of 88% on room air, blood pressure of 69/48 mmHg, no fever, and diminished air entry bilaterally more on the right side with rhonchi. Due to the shock state, initial intravenous (IV) normal saline 0.9% fluid resuscitation and high-flow mask oxygen were initiated in the ER, and the patient was admitted to the intensive care unit (ICU) for further management. IV fluid resuscitation was not successful enough and IV norepinephrine infusion was started in the ICU. Initial chest X-ray showed moderate right-sided pneumonia along with pleural effusion. Arterial blood gas showed severe metabolic and respiratory acidosis with a PaO2/FiO2 (P/F) ratio of 40 with worsening respiratory and mental status for the patient, and mechanical ventilation was started. The CT chest showed bilateral pneumonia with ARDS features as in Figure 1.

Blood, sputum, and pleural fluid cultures were negative, also autoimmune profiles including antineutrophil cytoplasmic antibodies (ANCA), rheumatoid factor (RF), and antinuclear antibodies (ANA) were done and came back negative. The echocardiography was normal. Only the C-reactive protein (CRP) and white cell count were mildly elevated. Supportive management was pursued for the first week as no infection could be proven.

**Figure 2:**
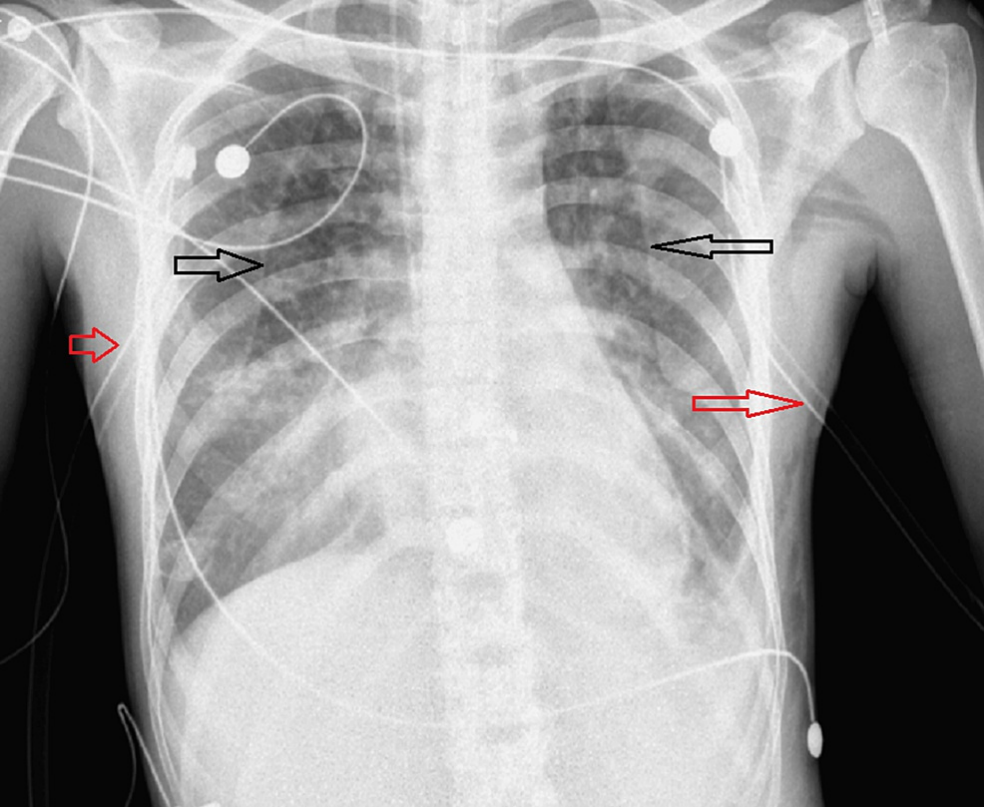
CXR with bilateral pneumothorax and chest tubes Black arrows point to the pneumothorax and red arrows point to the chest tubes. CXR: chest X-ray

During the second week, the patient developed bilateral pneumothorax on the routine chest X-ray, and the chest tube was inserted bilaterally as shown in Figure 2.

Then later on, the patient had a recurrence of the fever, tachycardia, and hypoxia. The blood workup was not significant, but the sputum culture detected ventilator-associated pneumonia (VAP) caused by carbapenem-resistant *Klebsiella *and IV and inhaled colistin was given.

On the third week of admission, the patient’s condition stabilized, but the weaning trial failed mostly due to ICU-related myopathy, rhabdomyolysis, and weakness; therefore, a tracheostomy was done, and the patient maintained on a ventilator in addition to excessive physical therapy and supportive management; later, the patient improved slowly, and he was successfully weaned from the mechanical ventilation with aggressive physical and occupational therapies. The patient was transferred to the medical floor after six weeks of ICU admission, then after two weeks, he was discharged from the hospital to rehabilitation with a plan for closing his tracheostomy opening after discharge on an outpatient basis.

## Discussion

Cannabis is a commonly used illegal drug with an increase in usage over the last decade. IDU, especially cocaine, amphetamine, and cannabis, has been associated with variable pulmonary complications like ARDS, diffuse alveolar hemorrhage (DAH), severe pneumonitis, and lung injury [4,5].

ARDS is a life-threatening condition that can complicate different pathologies like pneumonia, sepsis, trauma, pancreatitis, toxin inhalation, and IDU. IDU-related lung injuries could present with sudden onset of cough, hemoptysis, fever, and dyspnea. Some patients, however, present with severe hypoxemic respiratory failure requiring immediate ventilatory support with mechanical ventilation [6]. Our case was presented with the same clinical features of ARDS related to marijuana and amphetamine use which was confirmed by a careful history, excluding the other possible causes like infections and autoimmune diseases, and drug screening.

The effects of IDU on the lungs can be attributed to a variety of mechanisms. This damage can be either immediate after exposure such as acute lung injury, hypersensitivity reaction, or delayed such as reactive airway dysfunction syndrome [7]. All these pathologies occur due to the production of reactive oxygen species, activation of alveolar macrophages and neutrophils, and specific immune response [7].

The combination of cannabis and amphetamine can have a detrimental effect on the lung with DAH, pulmonary edema, and hemoptysis could occur as a possible complication, with no specific diagnostic criteria [8-12]. In rare cases, amphetamine and cannabinoids can cause rhabdomyolysis with unclear pathogenesis [13] which was observed in our patient and was managed conservatively.

Diagnosis of IDU-related lung injury is mainly clinical with exclusions of other possible causes and is supported by the medical history of drug use and positive drug screen; unfortunately, the management is supportive in most of the cases, with high mortality [10-12].

IDU continues to be a global health challenge with a range of associated complications. Pulmonary complications, such as lung injury, ARDS, and DAH are critical consequences of drug abuse and can lead to major morbidity and mortality with unclear pathogenesis. We report this case to highlight the need for early identification and intervention.

## Conclusions

The utilization of illicit drugs can lead to acute respiratory complications like ARDS, DAH, and pneumonitis with high mortality, necessitating a high level of suspicion among treating physicians in order to expedite the diagnosis and initiation of treatment to prevent further lung damage and improve patient outcomes. Further research is needed to clarify the precise mechanisms behind this association and to establish directed prevention and treatment strategies.
